# *Saccharomyces cerevisiae*: Population Divergence and Resistance to Oxidative Stress in Clinical, Domesticated and Wild Isolates

**DOI:** 10.1371/journal.pone.0005317

**Published:** 2009-04-24

**Authors:** Stephanie Diezmann, Fred S. Dietrich

**Affiliations:** Department of Molecular Genetics & Microbiology, Duke University Medical Center, Durham, North Carolina, United States of America; Washington University, United States of America

## Abstract

**Background:**

*Saccharomyces cerevisiae* has been associated with human life for millennia in the brewery and bakery. Recently it has been recognized as an emerging opportunistic pathogen. To study the evolutionary history of *S. cerevisiae*, the origin of clinical isolates and the importance of a virulence-associated trait, population genetics and phenotypic assays have been applied to an ecologically diverse set of 103 strains isolated from clinics, breweries, vineyards, fruits, soil, commercial supplements and insect guts.

**Methodology/Principal Findings:**

DNA sequence data from five nuclear DNA loci were analyzed for population structure and haplotype distribution. Additionally, all strains were tested for survival of oxidative stress, a trait associated with microbial pathogenicity. DNA sequence analyses identified three genetic subgroups within the recombining *S. cerevisiae* strains that are associated with ecology, geography and virulence. Shared alleles suggest that the clinical isolates contain genetic contribution from the fruit isolates. Clinical and fruit isolates exhibit high levels of recombination, unlike the genetically homogenous soil isolates in which no recombination was detected. However, clinical and soil isolates were more resistant to oxidative stress than any other population, suggesting a correlation between survival in oxidative stress and yeast pathogenicity.

**Conclusions/Significance:**

Population genetic analyses of *S. cerevisiae* delineated three distinct groups, comprising primarily the (i) human-associated brewery and vineyard strains, (ii) clinical and fruit isolates (iii) and wild soil isolates from eastern U.S. The interactions between *S. cerevisiae* and humans potentiate yeast evolution and the development of genetically, ecologically and geographically divergent groups.

## Introduction

*S. cerevisiae* has long been associated with humans as the fermentative agent in the production of bread, beer, and wine. Archeological evidence for the production of fermented beverages in China dates to 7,000 BCE, and molecular evidence demonstrating *S. cerevisiae* was the fermentative agent has been found in wine jars from ancient Egypt dating to 3,150 BCE [1], [2]. The close relationship between humans and yeast is further reflected in molecular signatures recovered from African artifacts that contained palm wine and from European wine and beer vessels that can be traced to Mesopotamia [3], [4]. Due to its close association with humankind, it has been speculated that yeast may have been the first living being domesticated [5]. Yet, the ecology of *S. cerevisiae* embraces a wider range than domesticated strains found in the vineyard and the brewery. Wild strains have been isolated from mushroom fruiting bodies as well as oak tree-associated soils and fluxes [6]–[11]. Wild isolates of *S. cerevisiae* are furthermore a major cause of spoilage of mango fruit and peach puree, and it has recently been identified in surveys of the fungal diversity in beetle guts [12]–[14].

The breadth of *S. cerevisiae* ecological diversity, encompassing domesticated and wild isolates, has spurred interest in the life history and population genetics of this species. The population biology of an organism so tightly associated with humans is challenging to study due to sampling bias, limited sampling sizes, human influence, and non-random sampling [5], [8], [15]. Consequently, questions concerning population structure and genetic diversity in *S. cerevisiae* have mainly been addressed using strains from grape berries, vineyards and other industrial applications. Fay and Benavides analyzed approximately 7 kb of coding and non-coding DNA sequences in 81 strains from vineyards, fermentation of sake, palm wine, ragi and cider, fruit sources, including lychee, fig, and mushrooms, oak tree and surrounding soil from New Jersey, and patients in the U.S. and Europe. This extensive analysis resulted in the recognition of domesticated, i.e. human-associated, and wild populations in *S. cerevisiae* [3]. Aa et al. examined 6.6 kb of coding and non-coding sequences from 27 strains including soil and oak-associated isolates from Pennsylvania, vineyard strains from two locations in Italy and strains from rotten figs from California [16]. The authors found signatures of distinct population structure, moderate levels of recombination and demonstrated that the oak isolates form a monophyletic group. In summary, previous studies indicated that ecology rather than geography coincided with population structure and that clinical isolates are only distantly related to isolates used in fermentation.

In addition to investigations into the origin and consequences of domestication, *S. cerevisiae*'s has been reported as an emerging opportunistic pathogen [17], [18]. Since the late 1950's, there have been increasing case reports of *S. cerevisiae* causing infections [19]. *S. cerevisiae* and its commercially available preparations known as *S. boulardii*, that are used to treat antibiotic-related diarrhea, have been shown to cause a wide variety of infections, ranging from cutaneous infections and vaginitis to systemic infections of the bloodstream and vital organs in immunocompromised and immunocompetent individuals [20]–[33]. These infections are similar to those by the related yeast *Candida albicans*, the most common human fungal pathogen [20], [34], [35]. Because of its evolutionary kinship with *C. albicans* and its status as genetics model system, *S. cerevisiae*, the benevolent baker's yeast, has acquired enhanced scientific value as a model pathogen in the study of virulence-related traits [36], [37].

Microbial virulence-related traits promote host invasion, colonization and virulence. One of these is the ability to survive oxidative stress, exerted by radical oxygen species (ROS), an integral component of mammalian host defenses, is associated with virulence in various bacteria [38]–[42]. Other virulence traits that have been studied in *S. cerevisiae* include growth at high temperature [43], the formation of multiple colony phenotypes [44], pseudohyphal growth [45], and loss of mitochondrial genome function [46]. These studies showed that clinical isolates differed phenotypically from laboratory and wine strains, but could not exclude the possibility that the observed association between clinical origin and a virulence trait is due to shared ancestry rather than host adaptation. If clinical isolates share a common ancestor, the evolution of virulence could be attributed to an isolated event that imparted selective advantage to one or more progenitor, pathogenic strains. However, if clinical isolates exhibit multiple evolutionary histories, pathogenicity would more likely reflect an adaptive advantage conferred by the acquisition of multiple virulence traits in different strains. This scenario is illustrated by the example of growth at 37°C, which is required for pathogenicity. It is teleologically reasonable to hypothesize that this trait is likely to be acquired by yeasts growing on fruit, which attain high temperatures during decomposition. This is supported by “heat death point” studies, which showed that *S. cerevisiae* isolates from spoiled peach pure and grape juice were heat resistant [13], [47]. Hence, clinical isolates could have acquired the ability to survive high temperatures via recombination with isolates from decomposing organic matter.

Despite progress in understanding the population structure of domesticated and wild *S. cerevisiae*, little is known about the emergence of this versatile yeast as a pathogen and the role of selection and/or adaptation in this evolutionary process. This study aims to (i) test for genetic differentiation between strains of a broad range of origins, (ii) investigate the evolutionary origin of clinical isolates, and (iii) identify an association between a virulence-related trait and pathogenicity. The targeted virulence trait is survival of oxidative stress. To investigate population structure and evolution of pathogenicity, an ecologically diverse sample of 103 *S. cerevisiae* isolates, comprising seven populations, was analyzed. Clinical isolates with confirmed virulence were obtained from the California Institute for Medical Research in Stanford and Duke University Medical Center in Durham, NC [43]. The domesticated, fermentation-associated, isolates consisted of brewery strains from Europe, and vineyard isolates collected from small commercial vineyards in from North Carolina and Australia (AU) [9], [48]. The wild strains consisted of fruit isolates, collected from tropical monocultures and fermenting fruits from different locales around the world, soil isolates from an arboretum in Pennsylvania (PA) and parks in North Carolina (NC) and insect guts from Louisiana. Commercial *S. boulardii* strains from France, Italy and Germany, complete the sampling [12], [33].

The results confirm previous findings and generated several novel conclusions. (i) At least three divergent groups representing different evolutionary trajectories were detected in *S. cerevisiae*, expanding the previous findings. (ii) Clinical isolates are genetically similar to the isolates from fruit, which supports the hypothesis that monoculture or fermenting fruits may serve as a natural reservoir for the evolution of clinical isolates. (iii) Strains from the clinic and Pennsylvania soil tolerate oxidative stress better than any other group. However, the clinical isolates are genetically diverse while the soil isolates are identical at all loci. This strongly suggests that resistance to ROS is an adaptive property of the clinical strains.

## Results

### All strains are diploid isolates of S. cerevisiae

All isolates in this study belong to the species *S. cerevisiae*, as confirmed by ITS sequencing and all strains were determined by FACS analysis to be diploid (data not shown). To ensure correct estimates of population genetic parameters haplotype phase within each locus was determined. For the majority of isolates the correct locus haplotype phase could be determined by visual analysis of sequence alignments. For 22 strains the correct phase of one ore more loci was identified using PHASE 2.1.1. [49], [50]. For ten of those haplotype phase assignments were verified by cloning and sequencing. Consequently, co-dominant marker assignments and two haplotypes per strain were used in the subsequent analyses. For calculations of population genetic parameters, strains sharing the same origin were grouped into populations. Seven populations were recognized including clinic, fruit, brewery, NC and AU vineyard, NC and PA soil.

### Within S. cerevisiae three genetically divergent groups are recognized

**Figure 1:**
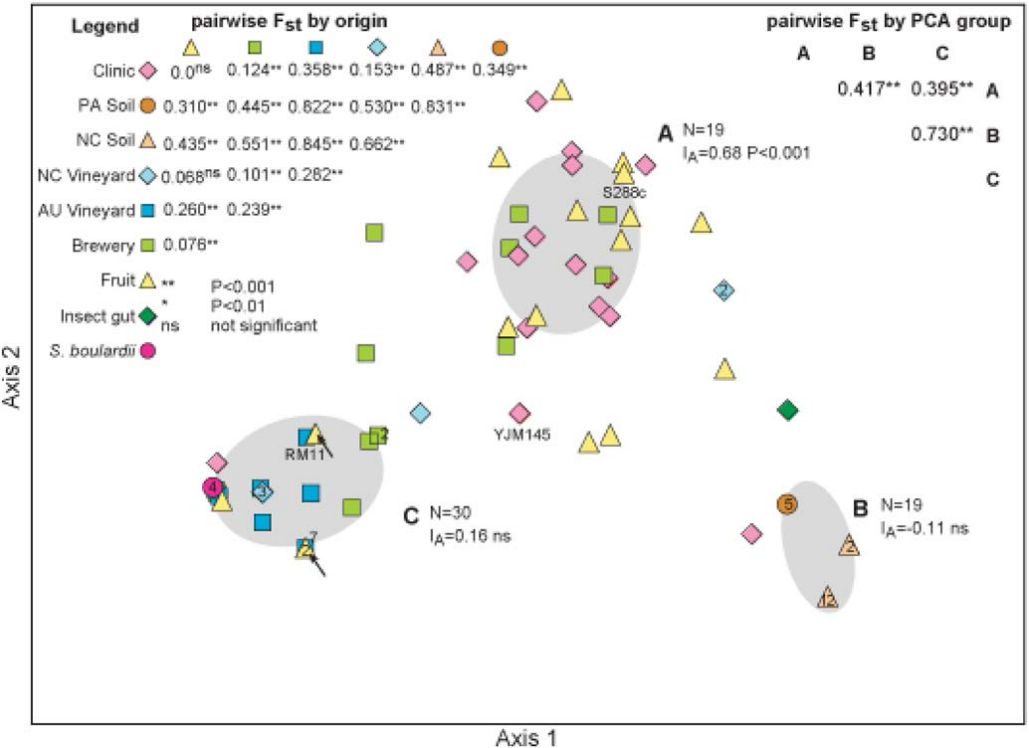
Three divergent groups in *S. cerevisiae*. PCA recognized three major groups (A, B, and C) in a combined analysis of complete sequence data for strains (N = 87) from all ecological backgrounds. The size (N) and index of association (I_A_), which is a measure of linkage equilibrium, of each group are given next to the group name. The I_A_ of each group has been calculated using sequence data for all five loci of each strain included in that group. Strain origins are coded with symbols and colors (see legend to the left). The arrows inside group C point to fruit isolates that have been isolated from grapes but are not part of the Australian or North Carolinian sampling. Strains of particular interest due to their history as lab strains (RM11, S288c) or importance as a model fungal pathogen (YJM145) have their names attached. Numbers inside symbols indicate how many strains share this genotype. Pairwise Fst values and significance levels for comparisons by origin (left) and PCA group (right) are indicated.

Principal component analysis (PCA), a transformation method that reduces multidimensional data sets to lower dimensions, was employed to detect putative structure among 87 isolates for which complete sequence data for five loci could be obtained (Table 1). Confidence envelope calculations of PCA results, revealed three major groups of 19, 19, and 30 isolates, each were identified and designated A, B, and C (Figure 1). The clustering was supported by UPGMA analysis (Unweighted Pair Group Method Arithmetic) of a genetic distance matrix derived from strains included in PCA (Figure S1). The composition of groups A, B, and C differs. The majority of clinical isolates (60%), a third of the fruit and brewery (33%) isolates are clustered in group A. All soil isolates from Pennsylvania and North Carolina comprise group B. All of the isolates from the Australian vineyards, half of the those from the North Carolina vineyards (50%), almost half the brewery isolates (42%), one-fourth of the fruit isolates (24%), one clinical isolate, and *S. boulardii* form group C. Note that two fruit isolates in group C stem from grapes, two fruit isolates from Holly and a papaya. Interestingly, all group C vineyard isolates have been collected from *Vitis vinifera* in Australia or North Carolina. The two identical NC vineyard isolates that are outside the major clusters were isolated from *V. rotundifolia* grapes, the Muscadine grapes that are native to the southeastern United States. Seven of the nine isolates collected in Adelaide (AU) are identical. As measure of genetic differentiation pairwise Fst values were calculated for groups A, B, and C. Fst values showed significant genetic differentiation between the three groups and analysis of molecular variance (AMOVA) indicated that 25% of the observed variance occurs within these groups and 62% between them. The index of association (I_A_), measuring the extent of linkage equilibrium, was significantly different from zero for group A but not for B and C.

**Table 1:** Origin, survival in oxidative stress and haplotype configurations of 103 strains included in this study.

Strain ID	Source	Survival in t-BH±SD^a^	*MLS1*	*ACT1*	*ADP1*	*PHD1*	*RPB1*
**CLINIC** (N = 15)							
YJM145	lung [25]	0.83±0.124	6/6^b^	1/1	1/1	1/1	1/1
MMRL124	human flank, DUMC^c^	0.64±0.284	3/5	1/3	1/1	5/5	3/3
MMRL125	human stool, DUMC	0.8±0.135	2/3	1/1	1/1	1/3	3/3
MMRL1620	Luzon, The Philippines [68]	0.74±0.163	1/3	1/7	4/4	1/5	1/1
MMRL2497	peritoneal dialysate, NC State lab	0.76±0.151	3/5	1/3	1/1	5/15	3/3
YJM273	[43]	0.78±0.09	1/6	1/1	1/6	1/12	1/3
YJM308	paracentesis fluid [69]	0.79±0.178	2/4	1/3	1/1	1/1	3/3
YJM309	blood [69]	0.77±0.016	3/6	1/1	1/1	2/2	3/3
YJM310	[43]	0.81±0.081	4/4	1/2	1/1	2/2	3/3
YJM311	bile tube [69]	0.89±0.031	1/3	1/1	1/4	8/19	1/3
YJM419	[43]	0.93±0.07	3/3	3/3	1/1	5/5	3/3
YJM434		0.74±0.049	2/2	2/3	1/4	2/2	1/1
YJM436	mouth [69]	0.79±0.121	3/3	1/1	1/1	1/1	3/3
YJM440	blood [69]	0.9±0.019	1/3	2/2	1/2	1/2	2/3
YJM454		0.77±0.136	7/7	1/1	1/1	3/3	2/2
**SOIL PA** (N = 10) [9]							
YPS128	*Q. alba*, soil	0.76±0.016	1/1	1/1	1/1	1/1	2/2
YPS129	*Q. alba*, flux	0.88±0.104	1/1	1/1	1/1	1/1	2/2
YPS133	*Q. alba*, soil	0.31±0.381	1/1	1/1	1/1	1/1	2/2
YPS134	*Q. velutina*, soil	0.89±0.047	1/1	1/1	1/1	1/1	2/2
YPS139	*Quercus* spp., soil	0.8±0.178	1/1	1/1	1/1	1/1	2/2
YPS141	*Q. velutina*, soil	0.78±0.107	1/1	1/1	1/1	1/1	ND^d^
YPS142	*Q. rubra*, bark	0.69±0.159	1/1	1/1	1/1	1/1	ND
YPS143	*Q. rubra*, soil	0.82±0.128	1/1	1/1	1/1	1/1	ND
YPS154	*Q. velutina*, bark	0.74±0.061	1/1	1/1	1/1	1/1	ND
YPS163	*Q. rubra*, soil	0.87±0.128	1/1	1/1	1/1	1/1	ND
**SOIL NC** (N = 14) (this study)							
O1	*Liriodendron tulipifera*, soil, OS	0.59±0.209	1/1	1/1	1/1	3/3	2/2
O2	*Q. prinus*, soil, OS	0.64±0.232	1/1	1/1	1/1	3/3	2/2
O3	*Gaultheria* spp., soil, OS	0.6±0.094	1/1	1/1	1/1	3/3	2/2
O4	*Q. prinus*, soil, OS	0.71±0.155	1/1	1/1	1/1	3/3	2/2
O6	*Acer* spp., soil OS,	0.75±0.068	1/1	1/1	1/1	3/3	2/2
O7	*Q. prinus*, soil, OS	0.61±0.082	1/1	1/1	1/1	3/3	2/2
O8	*Q. prinus*, soil, OS	0.45±0.07	1/1	1/1	1/1	3/3	2/2
O9	*Q. prinus*, soil, OS	0.59±0.05	1/1	1/1	1/1	3/3	2/2
SM1	*Q. alba*, soil, SM	0.46±0.07	1/1	1/1	3/3	3/3	2/2
SM2	*Q. alba*, soil, SM	0.58±0.084	1/1	1/1	3/3	3/3	2/2
SM12	*Acer* spp., soil SM	0.59±0.145	1/1	1/1	1/1	3/3	2/2
SM17	*Q. alba*, soil, SM	0.5±0.072	1/1	1/1	1/1	3/3	2/2
SM66	*Q. alba*, soil, SM	0.39±0.159	1/1	1/1	1/1	3/3	2/2
SM69	*Q. prinus*, soil, SM	0.43±0.063	1/1	1/1	1/1	3/3	2/2
**VINEYARD NC** (N = 10) [48]							
ARN019A	*Vitis vinifera* Chardonnay	0.37±0.083	2/2	1/3	1/4	2/2	1/1
ARN020A	*Vitis vinifera* Cabernet	0.37±0.113	2/2	1/3	1/4	2/2	1/1
ARN022A	*Vitis vinifera* Syrah	0.55±0.269	2/2	1/3	1/4	2/2	1/1
ARN056A	*Vitis vinifera* Riesling	0.7±0.09	2/2	3/3	1/5	1/2	2/4
ARN179A	*Vitis vinifera* Sangiovese	0.3±0.086	1/1	ND	1/1	3/3	ND
ARN202A	*Vitis vinifera* Syrah	0.38±0.211	1/1	ND	1/1	3/3	ND
ARN231A	*Vitis vinifera* Carlos	0.64±0.191	3/3	1/1	2/2	11/11	2/2
ARN239A	*Vitis vinifera* Carlos	0.25±0.08	1/1	ND	1/1	3/3	ND
ARN244A	*Vitis vinifera* Carlos	0.37±0.244	3/3	1/1	1/1	3/3	ND
ARN245A	*Vitis vinifera* Carlos	0.69±0.188	3/3	1/1	2/2	11/11	2/2
**VINEYARD AU** (N = 14) [48]							
ARC112A	*Vitis vinifera* Shiraz, Coonawarra	0.78±0.012	2/2	3/3	1/1	2/2	ND
ARA194B	*Vitis vinifera* white, Adelaide	0.26±0.083	2/2	2/3	1/1	8/8	1/1
ARS216A	*Vitis vinifera*, red, Sydney	0.96±0.038	2/2	1/3	1/1	2/2	1/4
ARS250B	*Vitis vinifera*, red, Sydney	0.91±0.038	2/2	1/2	1/4	2/2	1/1
ARS277B	*Vitis vinifera*, red, Sydney	0.3±0.132	2/2	3/3	1/1	2/2	1/1
ARA297A	*Vitis vinifera* Riesling, Adelaide	0.48±0.115	2/2	1/1	1/1	2/2	1/1
ARA299A	*Vitis vinifera* Shiraz, Adelaide	0.55±0.165	2/2	1/1	1/1	2/2	1/1
ARA306A	*Vitis vinifera* Shiraz, Adelaide	0.46±0.076	2/2	1/1	1/1	2/2	1/1
ARA315A	*Vitis vinifera* Shiraz, Adelaide	0.39±0.178	2/2	1/1	1/1	2/2	1/1
ARA316A	*Vitis vinifera* Shiraz, Adelaide	0.43±0.131	2/2	1/1	1/1	2/2	1/1
ARC364A	*Vitis vinifera* Shiraz, Coonawarra	0.03±0.024	2/2	2/2	1/1	2/2	1/1
ARA412A	*Vitis vinifera* Shiraz, Adelaide	0.6±0.157	2/2	1/1	1/1	2/2	1/1
ARA324A	*Vitis vinifera* Shiraz, Adelaide	0.47±0.084	2/2	1/1	1/1	2/2	1/1
ARA496A	*Vitis vinifera* Shiraz, Adelaide	0.29±0.202	2/2	1/3	1/1	2/2	1/1
**BREWERY** (N = 16)							
WY2124	Bohemian Lager	0.01±0.007	ND	2/2	1/6	4/4	ND
WY3787	Trappist	0.66±0.135	2/2	1/4	1/5	1/12	1/1
WY1026	British Cask Ale	0.45±0.099	2/9	1/2	2/9	4/10	3/7
WLP838	German Lager	0.04±0.029	ND	2/2	1/6	4/4	ND
WLP029	German Ale	0.03±0.034	ND	2/2	1/1	4/4	ND
WY3347	Eau de Vie	0.69±0.203	2/2	1/1	1/1	2/5	1/1
WY1388	Belgian Strong Ale	0.37±0.068	2/9	2/2	1/5	2/2	3/3
WLP033	English Ale	0.22±0.171	1/9	1/2	6/9	4/10	6/7
WLP775	English Cider	0.86±0.131	2/2	2/3	1/1	2/2	1/1
WLP036	Düsseldorfer Alt	0.43±0.071	9/14	1/2	1/9	5/8	1/3
WLP570	Belgian Golden Ale	0.24±0.052	2/9	1/2	1/5	2/2	1/3
WLP007	Dry English Ale	0.02±0.004	1/2	1/2	2/6	4/16	1/6
WLP099	High gravity	0.74±0.141	2/2	1/4	1/5	4/16	1/1
WY3632	Mead	0.59±0.178	2/2	1/4	1/5	1/10	1/1
WLP565	Belgian Saison I	0.61±0.261	9/9	1/1	1/1	ND	5/5
WY3184	Mead	0.56±0.3	1/9	1/2	6/6	4/10	3/7
**FRUIT** (N = 18)							
NRRL Y-35	*Ilex aquifolium*	0.04±0.044	2/2	3/3	1/1	2/2	1/1
NRRL Y-963	sour figs	1±0	3/3	1/1	1/1	2/2	2/2
NRRL Y-382	grain	0.37±0.079	5/9	1/1	1/2	1/17	3/9
NRRL Y-1537	grapes	0.27±0.081	2/2	1/1	1/1	2/2	1/1
NRRL Y-7568	rotten papaya	0.87±0.059	2/2	1/1	1/1	2/2	1/1
NRRL YB-210	spoiled banana	0.6±0.315	3/15	2/3	1/1	5/5	1/2
NRRL YB-4081	ripe guava	0.43±0.164	3/3	1/1	1/1	1/1	2/2
NRRL YB-4082	ripe papaya	0.39±0.194	5/5	1/1	1/1	1/1	3/3
NRRL YB-432	pineapple peal, Cuba	0.26±0.082	3/3	2/2	1/2	5/8	1/3
NRRL YB-908	wild cherry tree gum	0.69±0.201	11/11	5/5	1/1	13/13	8/8
NRRL Y-5511	coconut pod drippings	0.51±0.079	2/2	1/1	1/1	2/2	ND
NRRL Y-5997	ragi	0.84±0.119	1/1	1/1	6/6	7/7	3/3
NRRL Y-7662	pozol, Mexico	0.85±0.193	3/13	1/1	1/6	6/6	3/3
NRRL Y-11857	sugar refinery	0.9±0.098	1/3	1/1	2/2	2/2	3/3
NRRL Y-11878	cane juice, Jamaica	0.87±0.064	1/2	1/1	1/1	2/18	2/3
NRRL Y-12769	Malayan fermented tapioca	0.56±0.108	12/12	6/6	6/6	9/9	3/3
S344	diploid S288c, rotten fig [70]	0.15±0.062	3/3	1/1	1/1	12/12	3/3
RM11	fermenting grape must, Italy [71]	0.62±0.106	8/8	3/3	1/1	2/2	1/1
***S. BOULARDII*** (N = 4)							
Ysb1	Perenterol forte, this study	0.57±0.285	2/2	2/2	1/1	2/2	1/1
YJM1004	commercial [33]	0.18±0.133	2/2	2/2	1/1	2/2	1/1
YJM1005	commercial [33]	0.68±0.094	2/2	2/2	1/1	2/2	1/1
YJM1006	commercial [33]	0.52±0.138	2/2	2/2	1/1	2/2	1/1
**INSECT GUT** (N = 2) [12]							
IY 03-5-26-5-1-1	*Chauliodes rastricornis* (female), Livingston Parish, LA	0.71±0.035	ND	1/1	3/3	3/3	ND
IY 03-5-30-1-1-1	*C. rastricornis* (male), Livingston Parish, LA	0.86±0.033	3/3	1/1	3/3	3/3	2/2
Total Haplotypes d			198	200	206	204	176

Isolates are grouped by origin and sources as indicated. For each isolate the average survival in t-BP with one standard deviation are represented. Two haplotypes per locus and strain are summarized.

a standard deviation.

b GenBank accession numbers can be found in Table S1.

c Duke University Medical Center.

d no data.

### Levels of recombination, genetic diversity and linkage differ among S. cerevisiae populations

Seven populations encompassed a total of 82 strains for which complete sequence data were obtained. Five population genetics parameters concerning genetic differentiation (Fst), recombination, linkage equilibrium (I_A_), nucleotide diversity (π), and deviation from Hardy-Weinberg equilibrium (HWE) were calculated (Figure 1, Table 2).

**Table 2:** Nucleotide diversity (π), minimum number of recombination events, index of association (I_A_), and Hardy-Weinberg Equilibrium (HWE) for seven defined populations.

Origin	# of Strains	π×10^4^ (SD^a^)	Minimum # of recombination events^b^	I_A_	HWE^c^
Clinic	15	2.26 (0.18)	5	0.22^ns^	PHD1**, MLS1*, RPB1**
Soil PA	5	0.00 (0.00)	0	na	
Soil NC	14	0.3 (0.11)	0	na	ADP1***
Vineyard NC	6	2.18 (0.26)	1	3.05***	MLS1*
Vineyard AU	13	0.41 (0.11)	1	0.17^ns^	PHD1***
Brewery	12	2.64 (0.16)	5	0.67*	PHD1*
Fruit	17	2.82 (0.25)	5	0.32*	PHD1***, ACT1***, ADP1**, MLS1***, RPB1***
**Total**	**82**	**2.43 (0.09)**	**6**	0.81***	

Only complete data sets, totaling 82 strains, from seven different origins were included in the analysis.

a standard deviation.

b four gamete test.

c deviation from HWE calculated for each locus in each population.

na not applicable.

Significance values for I_A_ (***P<0.0001, **P<0.01, *P<0.05, ns not significant).

Significance values for HWE (***P<0.001, **P<0.01, *P<0.05) indicate significant deviation from HWE.

Genetic differentiation varied between populations from different origins (Figure 1). Clinical and fruit isolates were not significantly different from each other, but the clinical isolates were distinct from all other isolates. Fruit isolates differed from all except NC vineyard isolates. There was a large degree of genetic differentiation between AU vineyard and NC and PA soils (0.822, 0.845), and soil samples from NC and PA exhibited extensive genetic differentiation from each other (0.831). NC and AU Vineyard populations differed less from each other (0.282). The brewery population was most different from the soil populations (0.445, 0.551) and less different from vineyard and clinical populations (0.239, 0.101, 0.124).

Linkage equilibrium and the minimum number of recombination events calculated for each population differ between populations (Table 2). The I_A_ was significant for the NC vineyard, brewery and fruit populations. Each of the clinic, fruit and brewery populations could be explained by a minimum of five recombination events. The soil isolates showed no evidence of recombination and the vineyard isolates of only a single recombination event among these five loci. I_A_ and recombination results were reflected in the differences in nucleotide diversity between populations. Fruit, brewery, clinical, and NC vineyard populations exhibit high relative nucleotide diversity. Low π values were calculated for isolates from NC and PA soils and the AU vineyard population. π values are to a large degree in concordance with observed heterozygosity (Table 1). Between 33% and 88% of fruit, brewery, clinical and NC vineyard strains are heterozygous at one ore more loci. Indeed, four brewery isolates are heterozygous at every locus tested. NC and PA soil isolates are completely homozygous and 28% of AU vineyard isolates were heterozygous for at least one locus.

All populations were tested for deviations from HWE at each locus. A population that deviates from HWE does so because of non-random mating, mutation, and selection and other factors that affect population structure. Only the fruit population differs significantly at all loci from HWE (Table 2). The clinical population differs at three loci, NC soil, NC and AU vineyard, and brewery populations deviate at least at one locus. HWE calculations could not be conducted on the PA soil population.

### Clinical and domesticated isolates of S. cerevisiae exhibit haplotype diversity, soil isolates do not

**Figure 2:**
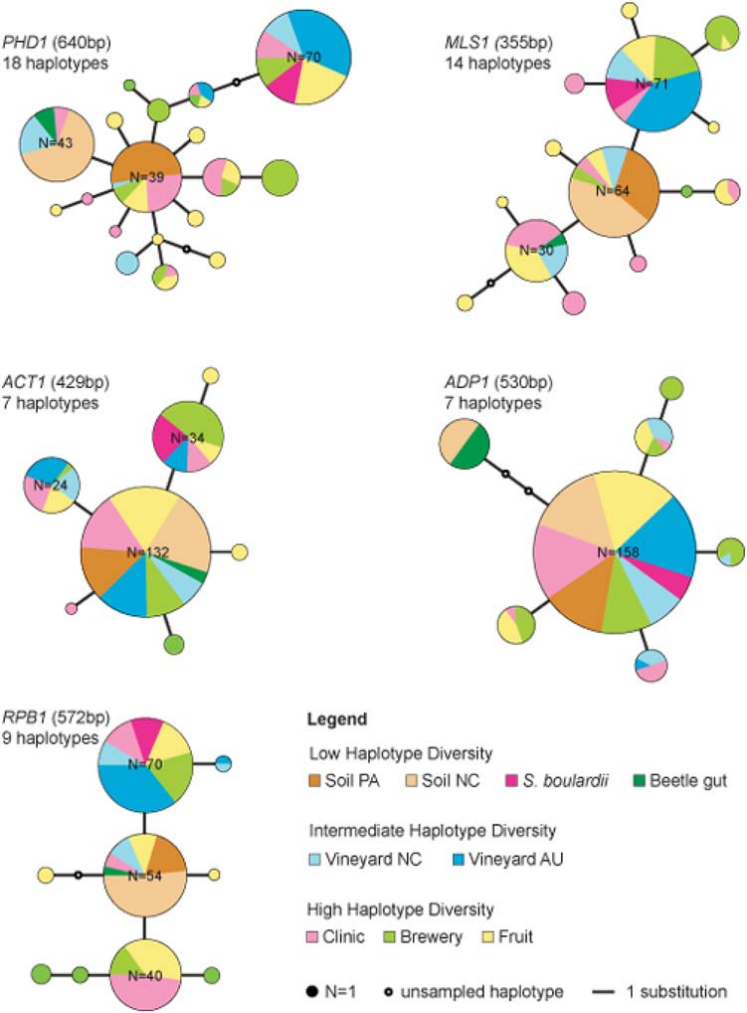
Haplotype diversity at five nuclear loci. Two haplotypes per strain were analyzed for five nuclear coding loci. Lengths of analyzed sequence data and number of sampled haplotypes are given for each locus. The size of each pie represents the number of identical haplotypes and the proportions indicate how many of those share a particular origin. If N>20, the number of haplotypes is indicated in the pie. The length of the connecting lines translates into nucleotide substitutions distinguishing one haplotype from another. Haplotypes collected from the clinic, the brewery and fruit sources are randomly distributed in each network, whereas soil isolates share one or two haplotypes. No correlation between strain origin and haplotype could be detected and no haplotype that unifies all strains from one origin.

Haplotype networks were inferred from the DNA sequence data of five nuclear coding loci, totaling 2,527 bp [51] (Figure 2, Table 1). Although haplotype diversity varied between loci, from 18 for *PHD1* to 7 for *ACT1* and *ADP1*, a common pattern was identified. Each network is characterized by the presence of one to three dominant haplotypes. Those accumulated mutations and gave rise to six to nineteen minor haplotypes in different networks, some of which have not been sampled in this study and are marked accordingly (Figure 2).

The haplotype diversity between populations differs widely, and three groups with different levels of diversity can be discerned. The soil populations are the least diverse. With two exceptions, all strains from NC and PA soils are represented by one of the dominant haplotypes in each network. In *ADP1*, NC soil isolates are represented by two haplotypes, and in *PHD1*, NC and PA soil isolates have different haplotypes. Both vineyard populations exhibit an intermediate amount of haplotype diversity – between two and four types per network. The clinical, brewery and fruit populations demonstrate the greatest diversity, in concordance with the population genetic parameters described above. They are represented by three to eleven different types in each network. Haplotypes from isolates of the same population do not cluster; for example, at the *MLS1* locus, clinical isolates originate from all three common haplotypes, not just one (Figure 2).

### Clinical and soil PA isolates of S. cerevisiae are highly resistant to oxidative stress

**Figure 3:**
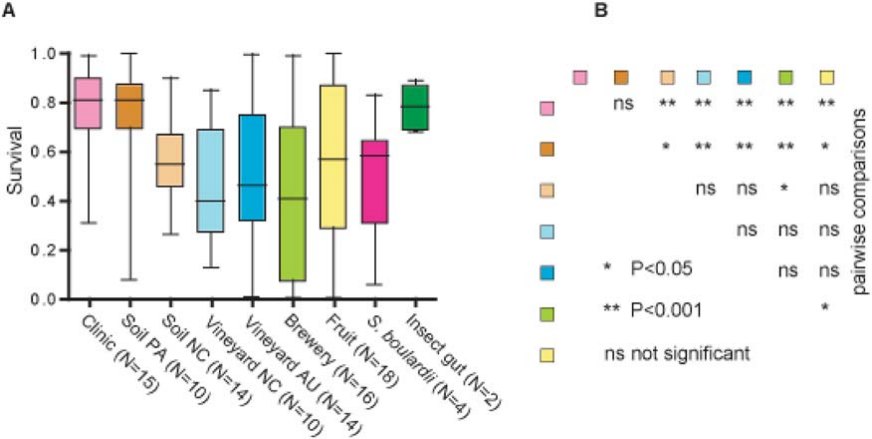
Survival of 103 *S. cerevisiae* strains in 20 mM TBHP. Box and whiskers plot for survival in 20 mM TBHP as tested in the CFU assay. Strains are clustered and color-coded by origin (Table 1). For each group of strains the colored box entails the size of the 25th and 75th percentile. The horizontal line dividing the box is the median (50^th^ percentile) and the whiskers represent the most extreme outliers with highest or lowest survival. Experiments were carried out in triplicates and all three values of each strain were used in this plot (A). The multiple pair-wise comparisons of all groups of N≥10 indicate that clinical isolates and strains from the soil in PA differ significantly from strains from other demographic groups. The legend denotes significance values obtained in ANOVA of all three experimental replicates of each strain (B).

All isolates were tested for survival of oxidative stress exerted by tert-Butyl hydroperoxide, a stable organic analog of H_2_O_2_. Clinical isolates, PA soil isolates, and strains from insect guts exhibited the highest mean survival rates (Figure 3A, Table 1). NC soil, both vineyard populations and the *S. boulardii* isolates show decreased survival and intermediate levels of variation. Brewery and fruit populations display survival rates ranging from very low to very high. Analysis of variance (ANOVA) confirmed significant differences in oxidative stress between groups of N≥10 (P<0.0001). Clinical isolates differ significantly (P<0.001) from all groups but PA soil (Figure 3B). PA soil isolates differ significantly (P<0.001) from the vineyard and the brewery isolates and only slightly (P<0.05) from the NC soil and fruit isolates. The remaining pair-wise comparisons were either not or only marginally significant (P<0.05).

## Discussion

Although *S. cerevisiae* is a species of inter-fertile isolates [9], [52], [53], PCA delineated three genetically distinct groups with different strain compositions. Group A, comprises isolates from genetically diverse and recombining populations collected in the clinic, the brewery and fruits. Group B includes the genetically homogenous but distinct PA and NC soil populations. Group C unites the distinct yet homogenous NC and AU vineyard isolates with genetically diverse brewery strains. These results are similar to the findings from the Fay and Benavides and Aa et al. studies where they reported that human association has shaped *S. cerevisiae* life history and led to domesticated isolates distinct from wild soil isolates [3], [16]. This study identified a third group that is dominated by clinical isolates and genetically different from domesticated and wild lineages. These three life history trajectories of *S. cerevisiae* are characterized by different levels of recombination and genetic differentiation that could explain their origin and maintenance.

Low levels of recombination and genetic diversity among soil populations (group B) suggest a predominantly clonal life style that contributes to differentiation from clinical and domesticated groups and maintenance of a wild lineage that also exhibits geographic structure because all of its isolates were collected in the eastern United States. Interestingly, the soil isolates share four of five haplotypes with beetle gut isolates, suggesting that insects acquire *S. cerevisiae* while foraging on the ground. In concordance with the Fay and Benavides results, ecology, rather than geography, appears to dominate evolution and origin of domestication in the vineyard and the brewery (group C) [3]. Although yeast isolates from NC and AU vineyards, collected from wine grapes, are genetically differentiated, they were recognized as most similar to each other and different from the Muscadine grape isolates in PCA. The importance of ecology is further emphasized by the observed distribution of brewery strains in PCA groups A and C, which suggests that brewery strains in wine-growing regions originated from grapes, and in areas lacking viniculture, the domesticated brewery yeasts were derived from wild fruit isolates.

The clinical and PA soil populations exhibited the highest mean resistance to ROS. Therefore ROS resistance in the clinical isolates arose either independently or was contributed by the soil isolates. The genetic diversity among the clinical isolates suggests multiple origins of the genetic material required for ROS resistance in these strains. Regardless, fungal resistance to ROS offers protection from oxidative host defenses and is undoubtedly an advantageous pathobiological property. Consistent with this conclusion, the probiotic strains of *S. boulardii* were not as resistant to ROS as the clinical strains. Indeed, strains of *S. boulardii* were less virulent compared to a clinical (YJM145) and a laboratory (Y55) strain [33]. The *S. boulardii* isolates used here were cultured from commercial products sold in France, Italy and Germany. Their close association with vineyard strains suggests that *S. boulardii* may have originated from vineyard strains.

Most recently, a study conducted by Kvitek, Will and Gasch combined investigations into genetic diversity with stress response and identified a distinct sake fermentation clade but no discrete clades for clinical or oak-associated isolates [54]. The discrepancy between the results reported by Kvitek et al. and our study can be explained by differences in strain sampling and number as well as the kind of assays conducted. The overlap in strain sampling is minimal. One PA soil and five clinical isolates were included in both studies. Furthermore, experimental procedures varied. While Kvitek et al. employed a plate-based assay, cells here were exposed to peroxide under defined conditions in liquid. Interestingly, strains isolated from similar environments share comparable stress response expression profiling patterns and oak isolates appear to have been selected for growth in this particular niche [54].

This investigation presents an exciting and provocative demonstration of the complex life history of *S. cerevisiae* beyond its prosaic service as a scientific tool in the laboratory or an agent of fermentation. As a species, *S. cerevisiae* entered two new life history trajectories while continuing its life in the soil and on decomposing fruit. Some strains of *S. cerevisiae* became domesticated in fermentation and brewing, and while others became pathogenic. With the accumulation of ecological and population genetic data, this versatile microbe becomes an invaluable model for evolutionary biology and population genetics. The current report offers a new paradigm for studying pathogenesis by identifying correlation(s) among the virulence traits of isolates and the ecology of their ancestral reservoirs. These correlations will identify the genotypes associated with pathogenic strains or the potential for pathogenicity and elucidate the evolution of pathogenicity.

## Materials and Methods

### Yeast strains, culturing and DNA extraction

*S. cerevisiae* strains were isolated from *S. cerevisiae* infected patients (N = 15), soil in Pennsylvania (PA, N = 10), soil in North Carolina (NC, N = 14), vineyards in NC (N = 10) and Australia (AUS, N = 14), various fruits (N = 18), brewery (N = 16), commercial *S. boulardii* preparations (N = 4), and the insect gut (N = 2) (Table 1). All strains were permanently stored at −80°C in 15% glycerol or cultivated on rich media plates (YPD, 1% **y**east extract, 2% **p**eptone, 2% **d**extrose, 2% agar). DNA was extracted using the CTAB buffer method [55]. NC soil samples were collected as part of this study using a previously established enrichment protocol and ITS sequencing for identification [9], [56].

### DNA sequencing and sequence analysis

Genomic DNAs were diluted 1∶100 and 2 µl added to a 25 µl reaction from TaKaRa Ex Taq kit with a final primer concentration of 0.6 µM (Table 3). The PCR regime consisted of 5 min initial denaturation at 95°C, followed by 35 cycles of 30 sec at 95°C, 30 sec at the appropriate annealing temperature (Table 3), and 45 sec at 72°C, concluding with 10 min extension at 72°C. PCR products were purified using the Montáge-SEQ_96_Cleanup Kit (Millipore) or the ExoSAP protocol and their concentration determined electrophoretically [57]. Between 500 and 1000 ng PCR product were sequenced with BigDye chemistry version 3 according to the instructions supplied by Applied Biosystems. Chromatograms were assembled and analyzed in Sequencher 4.8 (Gene Codes Corp.) and sequences edited in MacClade 4.06 [58]. ITS sequence data were blasted against GenBank using the nr database and the blastn algorithm [59].

**Table 3:** Primers used for amplification of nuclear loci and ITS.

Locus	Primer	Sequence (5′→3′)	Annealing temperature (°C)	Reference or source
*RPB1*	A_f_	GARTGYCCDGGDCAYTTYGC	50	[72]
	C_r_	CCNGCDATNTCRTTRTCCATRTA		
*MLS1*	MLS1F	TATGRCYGATTTTGAAGATT	50	This study
	MLS1R	TARTCCCAWCKWCCRCARTT		
*ACT1*	ACT1	TACCCAATTGAACACGGTAT	58	[37]
	ACT2	TCTGAATCTTTCGTTACCAAT		
*ADP1*	ADP1F	AATAAGTGGTATCGTGAAG	50	This study
	ADP1R	CTGACACTTTTTTGGCATTT		
*PHD1*	PHD1F	TCCCAGCCTATAACTTTGTGG	50	This study
	PHD1Fv2	CATGTTCCTGAAATGAGGCT		
	PHD1R	AGGAATCCAAACACCCTTGA		
ITS	ITS1	TCCGTAGGTGAACCTGCGG	53	[56]
	ITS4	TCCTCCGCTTATTGATATGC		

Shown are primer sequences that were used to amplify and sequence partial coding loci for population genetic analyses and the ITS for species confirmation.

The haplotype phase of nuclear loci in strains with more than one heterozygous site per locus was determined using PHASE 2.1.1. [49], [50]. Loci that received low confidence probabilities were cloned using the pCR2.1 TOPO TA Cloning Kit (Invitrogen) and sequenced as described above. Two haplotypes per strain were further analyzed. PCA, HWE, AMOVA, Fst, π, I_A_ were calculated in GenAlEx 6, DnaSP 4.50.3, and MultiLocus 1.3 [60]–[62]. 98.9% confidence envelopes were calculated for three PCA groups based on three times the standard deviation of the PCA scores of strains included in each cluster [63]. Strains with missing data points were excluded from these analyses and statistical significance values were calculated in 999 permutations (Fst), and 10,000 (I_A_). Due to limited sampling size *S. boulardii* and beetle gut isolates were not included in HWE, Fst, π and I_A_ calculations. An UPGMA tree based on a pair-wise genetic distances between isolates included in PCA was build in PHYLIP 3.68 [64]. Haplotype networks were inferred from *MLS1*, *ACT1*, *ADP1*, *PHD1* and *RPB1* in PAUP* 4.0b10 applying the parsimony criterion, conducting heuristic searches, and using tree-bisection-reconnection (TBR) as branch swapping algorithm. These five loci were chosen for analysis based on their prior employment in phylogenetic studies of the Saccharomycetales, population genetic analysis of *S. cerevisiae* and *C. albicans* and their differential expression patterns upon phagocytosis by macrophages (ACT1, RPB1 [37], ADP1 [65], MLS1 [66], PHD1 [16]).

### Survival during oxidative stress

Two-day old cultures from YPD plates were inoculated into liquid synthetic defined media (SD, 2% glucose, 37.8 mM (NH_4_)_2_SO_4_, 1.7 g/l yeast nitrogen base (YNB) without amino acids and ammonium sulfate), incubated at 30°C while shaking (250 rpm), transferred once, and grown over night. In order to reduce variability in the cell suspension, the cells were washed twice with 0.9% NaCl solution and dissolved in 1× phosphate buffered saline. After adjusting the cell number to 2×10^3^ cells/ml, 20 mM tert-Butyl hydroperoxide (TBHP, Sigma) were added and treated and control samples incubated for one hour at 30°C, while shaking. 100 µl cell suspensions from both samples were spread on YPD plates, incubated for 48 h at 30°C and colonies counted. Survival was calculated as the ratio of treated over untreated cells. Each strain was tested three times and results were clustered by origin and plotted in box-and-whisker plots. A one-way ANOVA with Bonferroni correction was carried out on experimental triplicates of each strain in every group to assess differences in variation for all groups with N≥10.

### Fluorescence-Activated Cell Sorting (FACS)

The ploidy of each *S. cerevisiae* strain was assessed using a previously published procedure with two modifications [67]. Exponentially growing cells were used and the final cell number was adjusted to 5×10^7^ cells/ml. The successful completion of the staining procedure was verified microscopically and cells were stored in the dark at 4°C until ready for FACS analysis. On average 9,860 gated events were measured per strain.

## Supporting Information

Table S1 Genbank accession numbers for haplotype sequences.
(0.04 MB DOC)

Figure S1 UPGMA tree. The tree was generated from a pair-wise genetic distance matrix based on haplotype data of the 87 strains that were included in PCA. The strains are color-coded by origin (legend) and groups recognized in PCA indicated at internodes in the tree. Strains marked with * denote isolates that are not included in PCA groups A, B or C confidence envelopes. The numbered bar below the tree indicates total genetic distance observed in the data set.
(1.34 MB TIF)
