# Supplementary material for: Saccharomyces cerevisiae: Population Divergence and Resistance to Oxidative Stress in Clinical, Domesticated and Wild Isolates
Source: PLoS One. 2009 Apr 24;4(4):e5317. doi: 10.1371/journal.pone.0005317 (PMC2669729; doi:10.1371/journal.pone.0005317)
Supplement: Table S1 — Genbank accession numbers for haplotype sequences. (0.04 MB DOC) [file pone.0005317.s001.doc]

**Table S**1: GenBank accession numbers for haplotype sequences.

| Haplotype | *MLS1* | *ACT1* | *ADP1* | *PHD1* | *RPB1* |
| --- | --- | --- | --- | --- | --- |
| 1 | FJ787003 | FJ786989 | FJ786996 | FJ787017 | FJ787035 |
| 2 | FJ787004 | FJ786990 | FJ786997 | FJ787018 | FJ787036 |
| 3 | FJ787005 | FJ786991 | FJ786998 | FJ787019 | FJ787037 |
| 4 | FJ787006 | FJ786992 | FJ786999 | FJ787020 | FJ787038 |
| 5 | FJ787007 | FJ786993 | FJ787000 | FJ787021 | FJ787039 |
| 6 | FJ787008 | FJ786994 | FJ787001 | FJ787022 | FJ787040 |
| 7 | FJ787009 | FJ786995 |  | FJ787023 | FJ787041 |
| 8 | FJ787010 |  |  | FJ787024 | FJ787042 |
| 9 | FJ787011 |  | FJ787002 | FJ787025 | FJ787043 |
| 10 | a |  |  | FJ787026 |  |
| 11 | FJ787011 |  |  | FJ787027 |  |
| 12 | FJ787013 |  |  | FJ787028 |  |
| 13 | FJ787014 |  |  | FJ787029 |  |
| 14 | FJ787015 |  |  |  |  |
| 15 | FJ787016 |  |  | FJ787030 |  |
| 16 |  |  |  | FJ787031 |  |
| 17 |  |  |  | FJ787032 |  |
| 18 |  |  |  | FJ787033 |  |
| 19 |  |  |  | FJ787034 |  |

^a^ Gaps indicate haplotypes that were identified but not included in the final analyses presented here.
