# Supplementary figures and images for: Saccharomyces cerevisiae: Population Divergence and Resistance to Oxidative Stress in Clinical, Domesticated and Wild Isolates
Source: PLoS One. 2009 Apr 24;4(4):e5317. doi: 10.1371/journal.pone.0005317 (PMC2669729; doi:10.1371/journal.pone.0005317)

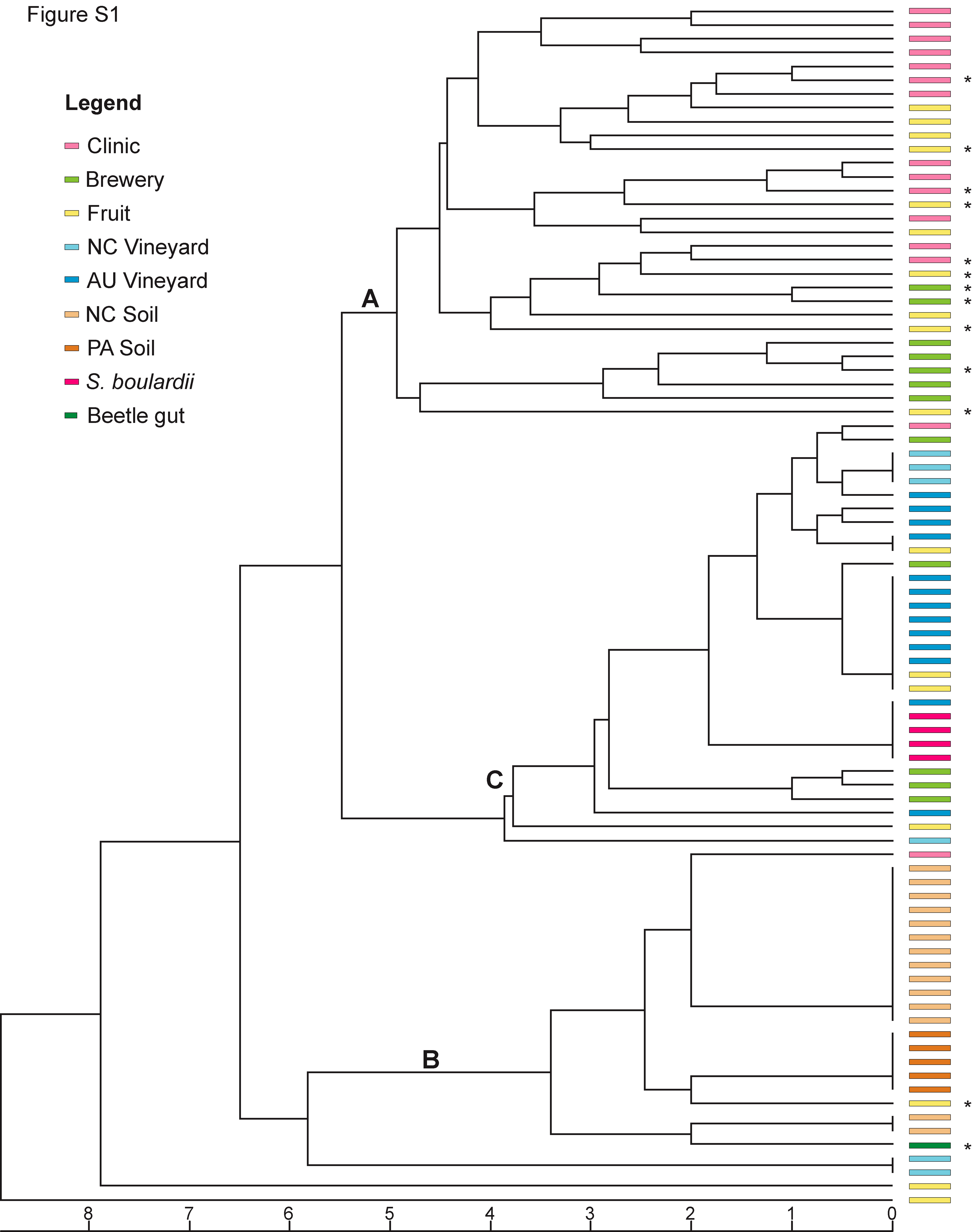

Supplement: Figure S1 — UPGMA tree. The tree was generated from a pair-wise genetic distance matrix based on haplotype data of the 87 strains that were included in PCA. The strains are color-coded by origin (legend) and groups recognized in PCA indicated at internodes in the tree. Strains marked with * denote isolates that are not included in PCA groups A, B or C confidence envelopes. The numbered bar below the tree indicates total genetic distance observed in the data set. (1.34 MB TIF) [file pone.0005317.s002.tif]
